# Host specificity and the structure of helminth parasite communities of fishes in a Neotropical river in Mexico

**DOI:** 10.1051/parasite/2016073

**Published:** 2016-12-22

**Authors:** Guillermo Salgado-Maldonado, María Teresa Novelo-Turcotte, Juan Manuel Caspeta-Mandujano, Gabriela Vazquez-Hurtado, Benjamin Quiroz-Martínez, Norman Mercado-Silva, Mario Favila

**Affiliations:** 1 Universidad Nacional Autónoma de México, Instituto de Biología, Departamento de Zoología, Laboratorio de Helmintología Apartado Postal 70-153 C.P. 04510 Ciudad de México Mexico; 2 Universidad Autónoma del Estado de Morelos, Facultad de Ciencias Biológicas y Centro de Investigaciones Biológicas, Laboratorio de Parasitología de Animales Silvestres Avenida Universidad Número 1001, Colonia Chamilpa C.P. 62209 Cuernavaca Morelos Mexico; 3 Instituto de Ecología A. C. Carretera antigua a Coatepec 351, El Haya Xalapa 91070 Veracruz Mexico; 4 Universidad Autónoma del Estado de Morelos, Centro de Investigación en Biodiversidad y Conservación Avenida Universidad Número 1001, Colonia Chamilpa C.P. 62209 Cuernavaca Morelos Mexico

**Keywords:** Freshwater fish helminths, Sharing of helminths, Autogenic/allogenic dichotomy, Indices of host specificity

## Abstract

In a tropical locality of Río La Antigua, Veracruz, Mexico, 11 fish species, represented by 244 individual fish from six freshwater fish families living sympatrically and synchronically, were examined for helminth parasites. A total of 36 taxa of helminths were recorded, 24 autogenic and 12 allogenic forms, including 6 monogeneans, 14 trematodes, 1 cestode, and 15 nematodes. Most helminth taxa were recovered for 10/11 of the component communities we analyzed. The results contribute empirical evidence that host specificity is an important force in the development of helminth communities of freshwater fishes. Each fish family has their own set of parasites, host species belonging to the same taxon share parasite species. High component community similarity among related host species was recorded, demonstrated by high prevalence and abundance, as well as dominance, of autogenic specialist species in each component community. Most autogenic helminth species are numerically and reproductively successful in relatively few host species. Autogenic helminths common in one host species are not common in others. Our findings give empirical support to the idea that low levels of sharing of parasites favor animal coexistence and high species richness, because large phylogenetic differences allow potentially competing animals to consume the same resources without being sensitive of another’s parasites.

## Abstract (fr)

Dans une localité tropicale de Río La Antigua, Veracruz, au Mexique, onze espèces de poissons, représentées par 244 individus appartenant à 6 familles de poissons d’eau douce vivant en sympatrie et en synchronie, ont été examinées pour détecter les helminthes parasites. Un total de 36 taxons d’helminthes ont été trouvés, 24 autogènes et 12 allogènes, dont 6 monogènes, 14 trématodes, 1 cestode et 15 nématodes. La plupart des taxons d’helminthes ont été trouvés dans 10/11 des communautés de composants que nous avons analysées. Les résultats apportent une preuve empirique que la spécificité à l’hôte est une force importante dans le développement des communautés d’helminthes des poissons d’eau douce. Chaque famille de poissons a son propre ensemble de parasites et les espèces d’hôtes appartenant au même taxon partagent des espèces de parasites. Une similarité élevée des composants de la communauté parmi les espèces liées a été enregistrée, et ceci a été démontré par la prévalence et l’abondance élevées, ainsi que la dominance, des espèces autogènes spécialisées dans chaque communauté de composantes. La plupart des espèces d’helminthes autogènes ont un succès numérique et reproductif chez un relativement faible nombre d’espèces d’hôtes. Les helminthes autogènes communs dans une espèce hôte ne sont pas fréquents dans d’autres. Nos résultats fournissent un soutien empirique à l’idée que des faibles niveaux de partage des parasites favorisent la coexistence des animaux et une richesse élevée en espèces, parce que des grandes différences phylogénétiques permettent aux animaux potentiellement concurrents de consommer les mêmes ressources sans être sensibles aux parasites d’un autre.

## Introduction

Host specificity strongly influences the biogeography of parasites. This factor will determine the likelihood that a parasite will successfully invade a new habitat or adjust to new hosts following its arrival in new geographic areas [23, 26]. The close relationship between parasites and their hosts, and between hosts and their environment, are determinants of the character of regional helminth fauna [7, 9, 30, 38]. Each fish family commonly seems to have a set of parasites that are exclusive to and widely distributed in the host family, with limited host-sharing among them. This pattern has been documented in northern temperate fishes [5, 7, 35] as well as in tropical Mexican freshwater fishes [2, 6, 22, 28–31, 37]. As a result, host specificity appears to be an important factor in the distribution of parasites, since the distribution of helminths reflects that of the fish families they parasitize [3, 29–31]. This concept is widely accepted; however, few if any investigations have been conducted in order to test this tenet in a given host assemblage.

When analyzing the structure of helminth communities, allogenic/autogenic species distinction seems to be necessary since both kinds of species play different roles in the community and follow different ecological and evolutive routes [8, 11, 15]. The relationship between autogenic helminth species and their definitive fish host is a strong one: autogenics remain for a relatively lengthy period on or within fish tissues/organs, derive from them habitat facilities, food, dispersal capabilities, and significantly, a place for maturation and reproduction, i.e. a place to mate and allow genetic exchange. Whereas allogenic species are often opportunistic, they are not strongly linked to any fish host. They tend to use any fish species available when arriving in a novel locality [11]. Allogenic species do not grow up to reproduce in fish. They act as host generalists in fish because these hosts are indeed trophic channels to the final host [1]. Spreading individual parasites in as many small fishes as possible, with no regard for fish species, arguably will improve opportunities to reach more suitable bird definitive hosts, regardless of bird size. Contrarily, autogenic species need to face an effective reproduction and genetic exchange in definitive fish hosts, and then they concentrate individuals in selected hosts, restricted by the nature of their transmission and then physiological compatibility. Their more intimate interaction with the host restricts their host range more strongly. Autogenic species need to avoid host defences and to resist these for a longer time for reproduction of the parasite. In such a way that the composition of autogenic helminth species of fish will depend directly on the icthyological composition of the locality and will constitute an integral and consistent part of the community. Contrarily, the composition of allogenic species may depend on the geographical position of the body of water, as related to, for example, bird migration routes, and will be less predictable and not a consistent component of the parasite community [11, 14, 15].

Tropical fish assemblages can be very rich. In these sympatric and synchronic assemblages, helminth parasites could be equally available for most fishes such that generalist species of helminths would be numerous. However, because of host specificity to family level, each family of fishes would have their own parasites, meaning specialist species would be present in an important number. Addressing this concept is important in order to explore local factors such as sympatry and synchrony of host species, and their influence over regional distributions. With this in mind, in order to explore the role of host specificity in structuring helminth communities of fishes in a tropical locality, in this work we focus on helminth communities and sharing between 11 sympatric and synchronic species of freshwater fishes in a Mexican river.

## Materials and methods

We gathered data from a single sample of fishes caught on November 20, 2009, from Arroyo Apazapan (19°19′32″ N, 96°43′33″ W, altitude 294 m) in the eastern Eje Neovolcánico de México (Trans Mexican Volcanic Belt), a Neotropical locality belonging to the middle basin of Río La Antigua, near Xalapa, Veracruz, about 100 km from the mouth of the Río La Antigua opening into the Gulf of Mexico. This locality is inhabited by 11 sympatric fish species [19, 20]. In order to collect enough individuals (≈30 specimens) of each fish species to examine them for helminths, we sampled the main habitat types from a selected 200 m, ≈8 m wide, and 60 cm mean depth stream section. Substrate was composed mainly of sandy slime floor mostly covered by rocks. Fish were captured using an electrofishing device, transported live to the laboratory and immediately examined for helminths, within 24 h post capture. Tissues and organs of every single fish were searched for helminths under stereomicroscopes [see 34]. Two kinds of data were collected from each individual fish: the number of helminth species in each fish and the number of helminth individuals per helminth species (the abundance distribution). Voucher specimens of each helminth taxon were deposited in the Colección Parasitológica de la Universidad Autónoma del estado de Morelos (COPAUAEM), Facultad de Ciencias Biológicas, Laboratorio de Parasitología de Animales Silvestres, Universidad Autónoma del Estado de Morelos, Cuernavaca, Morelos, Mexico.

Data analyses were made at the component community level [12], i.e. all helminths in all individuals of each fish species examined in the locality. Sampling adequacy for all component communities was evaluated using randomized (100×) sample-based species accumulation curves computed in EstimateS (version 8.0 RK Colwell, http://viceroy.eeb.unconn.edu/estimates). For each component, we examined the asymptotic richness based on Clench’s model equation [36], as well as the final slope of the randomized species accumulation curve [13], that is, the gradient between the final two sampling points. A final value for the slope of the species accumulation curve no higher than 0.1 species per sample was used as the criterion for adequate sampling. Empirically, this final slope for the accumulation curve indicates that at least 70% of the species in the component have already been sampled [13]. Clench’s model is described by the following function:

V2=(a×V1)/(1+(b×V1))

where *V*2 is the observed richness, *V*1 is the number of hosts examined, and *a* and *b* are parameters of the curve; *a* equals the rate of adding new species and *b* is a parameter related to the shape of the curve. These values were calculated iteratively using EstimateS and Statistica software, as in [13]. The slope of the cumulative species curve was calculated as *a*/(1 + *b × n*)^2^ where *a* and *b* are the above parameters and *n* is the number of hosts examined from a given component community. Clench’s model equation allows estimation of the total number of species in a component as *a*/*b*.

In order to answer the question of how many species are likely to have been missed by inadequate sampling, we estimated the number of rare species missing from each component community using the Bootstrap nonparametric richness estimator (*S*_b_) [18, 24, 25]:

$$S_{b}=S_{o}+\sum \left[{1-(h_{j}/H)}^{H}\right]$$

where *S*_o_ is the observed species richness, *H* is the number of host individuals sampled from the component community, and *h*_*j*_ is the number of host individuals in the sample in which parasite species *j* is found. This also allows us to corroborate the richness estimation obtained using Clench’s model.

To qualitatively estimate the share of helminth species among sympatric host species (i.e. to assess the similarity between component communities in terms of the helminth species they support), we compared the composition of all component communities by means of Sørensen’s index [18]. Sørensen’s metric was used to compare samples of nearly equal size from most component communities, to weight matches in species composition between component communities (more heavily than mismatches), and because we wished to weight rare species the same as common species.

We measured host specificity of each helminth species in an effort to estimate the breadth of their ecological niche. We calculated a taxonomic distinctness metric used as a host-specificity index, *S*_TD,_ [26], to measure the strength of phylogenetic relationships among the host species, taking into consideration an evolutionary perspective. We calculated *S*_TD_ as a measure of the average taxonomic distinctness of all host species used by a species of parasite. The lowest value that this index can take is 1, when all host species are congeners, and the maximum value is 5, when all host species belong to different classes [26]. To account for the relative importance of different host species as resources for parasites in the studied locality, we also calculated the host-specificity index *S*_TD_* [27]. Here, the average taxonomic distinctness among the host species used by a parasite is weighted for the parasite’s prevalence in the different hosts. The value of *S*_TD_* is inversely proportional to specificity, a high index value means that the host species more frequently used by a parasite are not closely related [27].

Allogenic and autogenic helminth species as defined in [10] were distinguished. Autogenic species complete their life cycles in water and are incapable of crossing land barriers between freshwater bodies; allogenic species use fish only as intermediate or paratenic hosts and mature in vertebrates other than fishes, generally birds or mammals.

## Results

A total of 244 individual fish hosts, including all the 11 fish species living sympatrically in Arroyo Apazapan, were examined. Thirty-six helminth taxa, including eight adult trematodes, six metacercariae, six monogeneans, one adult cestode, nine adult nematodes, and six nematode larvae (Table 1), were recorded. One to ten helminth species were observed in the component communities (Table 2). Sampling effort was adequate for the inventory of helminth species for most of the component communities analyzed, except for the gobiid *S. gymnogaster*. For the 10 other fish species, the slope of the last point of each curve calculated from Clench’s model was ≤0.1 (Table 2), meaning that the observed species richness was no less than 80% of the real number of species in each component community. Estimation of richness by means of the Clench’s model parameters as well as the values of the Bootstrap nonparametric estimator, *S*_b_, confirms the patterns of richness observed (Table 2). However, the analysis of richness suggested that at least six rare helminth species from Apazapan fishes went undetected. We recorded nine helminth species of *Astyanax mexicanus* and the analysis suggested than two more species should have been recorded. Ten species were observed in *Vieja fenestrata* but both Clench’s model and the Bootstrap nonparametric estimator suggested the existence of two more unrecorded species. While 10 helminth species were recorded from *Rhamdia guatemalensis* and seven from *Poecilia sphenops*, the analysis suggested that at least one more species was missing from each host species to complete the inventory. This means that we detected 85.7% (36 of 42) of the species of helminths in Apazapan. In fact, the number of undetected species may be less than six because some of the species missing from different component communities could be the same.

**Table 1.** Prevalence %, and abundance of 36 taxa of helminth parasites of 11 sympatric fish species from Arroyo Apazapan, Río La Antigua, Veracruz, Mexico, November 2009.

Hosts examined	*A*. *mexicanus*	*S*. *gymnogaster*	*R*. *guatemalensis*	*H*. *bimaculata*	*P*. *mexicana*	*P*. *sphemops*	*P*. *gracilis*	*X*. *helleri*	*T*. *ellioti*	*V*. *fenestrata*	*S*. *marmoratus*
	18	6	30	29	28	31	30	20	26	19	7
*Culuwiya cichlidorum* AU (COPAUAEM T-460)					78.6%, 3.5	83.9%, 5.03	100%, 5.6	50.0%, 1.4		26.3%, 0.78	
*Genarchella isabellae* AU (COPAUAEM T-461)			10.0%, 0.26								71.4%, 1.57
*Auriculostoma astyanace* AU (COPAUAEM T-462)	5.5%, 0.11										
*Paracreptotrematoides heterandriae* AU (COPAUAEM T-463)				58.6%, 1.24							
*Wallinia chavarriae* AU (COPAUAEM T-464)	11.1%, 0.33										
*Magnivitellinum simplex* AU (COPAUAEM T-465)	5.5%, 0.11										
*Crassicutis cichlasomae* AU (COPAUAEM T-466)									50.0%, 1.19	57.9%, 1.78	
*Phyllodistomum inecoli* AU (COPAUAEM T-467)				6.9%, 0.06							
*Clinostomum* cf. *marginatum* AL (COPAUAEM T-468)			30.0%, 1.26			6.4%, 0.09			11.5%, 0.11	5.3%, 0.05	
*Ascocotyle nana* AL (COPAUAEM T-469)				58.6%, 13.41							
*Ascocotyle felippei* AL (COPAUAEM T-470)					7.1%, 0.17	3.2%, 0.09	6.7%, 0.16	10.0%, 0.1			
*Centrocestus formosanus* AL, i (COPAUAEM T-471)	77.7%, 40.22	16.6%, 0.16	90.0%, 87.5	51.7%, 4.13	39.3%, 1.92	19.3%, 0.58	63.3%, 3.83	85.0%, 25.15	26.9%, 0.76	5.3%, 0.05	71.4%, 17.57
*Posthodiplostomum minimum* AL (COPAUAEM T-472)					10.7%, 0.28	12.9%, 3.16		5.0%, 0.05			
*Uvulifer ambloplitis* AL (COPAUAEM T-473)	16.7%, 0.33				14.3%, 0.25	25.8%, 1.41	40.0%, 4.36	15.5%, 0.6	15.4%, 1.57	5.3%, 1.68	
*Aphanoblastella travassosi* AU (COPAUAEM M-101)			20.0%, 0.53								
*Sciadicleithrum mexicanum* AU (COPAUAEM M-102)										52.6%, 1.52	
*Urocleidoides strombicirrus* AU (COPAUAEM M-103)	88.9%, 6.27										
*Urocleidoides* cf. *vaginoclaustrum* AU (COPAUAEM M-104)				44.8%, 1.65				65.0%, 1.75			
*Gyrodactylus* sp.^AU^ (COPAUAEM M-105)	11.1%, 0.11										
*Gyrodactylus bullatarudis* AU, i (COPAUAEM M-106)					32.1%, 1		6.7%, 0.83				
*Bothriocephalus* cf. *cuspidatus* AU (COPAUAEM C-100)			13.3%, 0.26								
*Freitascapillaria moraveci* AU (COPAUAEM N-575)				34.5%, 0.75							
*Procamallanus neocaballeroi* AU (COPAUAEM N-576)	33.3%, 0.38										
*Procamallanus* sp.^AU^, * (COPAUAEM N-577)										5.3%, 0.05	
*Cucullanus angeli* AU (COPAUAEM N-578)										5.3%, 0.05	
*Cucullanus mexicanus* AU (COPAUAEM N-579)			66.7%, 1.17								
*Spinitectus mexicanus* AU (COPAUAEM N-580)				27.6%, 0.41							
*Rhabdochona kidderi* AU (COPAUAEM N-581)			3.3%, 0.1						80.8%, 3.69	52.6%, 1.68	
*Rhabdochona mexicana* AU (COPAUAEM N-582)	5.5%, 0.05										
*Capillaria* sp.^AU^ (COPAUAEM N-583)			3.3%, 0.03								
*Contracaecum* sp.^AL^ (COPAUAEM N-584)			3.3%, 0.03		3.6%, 0.1		3.3%, 0.23				28.6%, 0.28
*Hysterothylacium* sp.^AU^, ^L^ (COPAUAEM N-585)						3.2%, 0.09					
*Cucullanus* sp.^AU^, ^L^ (COPAUAEM N-586)										10.5%, 0.15	
*Spiroxys* sp.^AU^, ^L^ (COPAUAEM N-587)			6.7%, 0.1								
*Rhabdochona* sp.^AU^, ^L^ (COPAUAEM N-588)				6.9%, 0.06							
Acuaridae gen. sp.^AL^ (COPAUAEM N-589)									3.8%, 0.03		

AU Autogenic;

AL Allogenic;

L Larvae;

i Introduced species; between parentheses catalog numbers of voucher specimens of each helminth taxa deposited at COPAUAEM.

**Table 2.** Summary of the richness analysis and parameters of the cumulative curves of species for 11 component communities of helminths of freshwater fishes from Río Apazapan, basin of Río La Antigua, Veracruz, Mexico (in all cases the correlation coefficient *R*^2^ between observed data and Clench’s model > 0.96).

Host species	No. of hosts examined (*n*)	No. of helminth species (*S*_obs_)	Parameters of Clench		Richness estimated by Clench model *a*/*b*	Slope Clench *a*/(1 + *b × n*)^2^	Bootstrap nonparametric estimator *S*_b_
			*a*	*b*			
*A*. *mexicanus*	18	9	2.212358	0.198432	11.1	0.1058	10.35
*S*. *gymnogaster*	6	1	–	–	–	–	–
*R*. *guatemalensis*	30	10	1.964535	0.170238	11.5	0.0526	11.26
*P*. *bimaculata*	29	8	4.684552	0.550558	8.5	0.0162	8.25
*P*. *mexicana*	28	7	1.971636	0.249048	7.9	0.0310	7.54
*P*. *sphenops*	31	7	1.289638	0.157334	8.1	0.0373	7.86
*P*. *gracilis*	30	6	1.861581	0.291741	6.4	0.0195	6.25
*X*. *helleri*	20	6	2.784144	0.427796	6.5	0.0304	6.51
*S*. *marmoratus*	7	3	3.685704	1.055253	3.4	0.0523	3.09
*T*. *ellioti*	26	6	2.236039	0.339503	6.5	0.0231	6.41
*V*. *fenestrata*	19	10	2.020171	0.160467	12.6	0.1232	11.91

Twelve of the 36 recorded taxa were larval forms, eight of which were allogenic species including six metacercariae and two nematode larvae that mature in bird hosts (Table 1). Most allogenic species are generalists as they infect several unrelated host species. For example, the introduced metacercariae of *Centrocestus formosanus* were recorded from all 11 fish species, reaching a prevalence >20% in nine of these species. The remaining four larval or immature nematodes, including *Hysterothylacium* sp., *Cucullanus* sp., *Spiroxys* sp., and *Rhabdochona* sp., are autogenic species, as are the 24 adult helminths recorded (Table 1).

Each host species or related host taxon harbors a particular assemblage of autogenic species; most autogenic species are successful numerically in few host species, such that helminths common in one host species are not common in others (Table 1). Autogenic helminths in Apazapan are mostly specific to a single host species, or they exploit a narrow taxonomic range of hosts, infecting species belonging to the same family. Limited sharing of autogenic species was recorded. The monogeneans *Urocleidoides vaginoclaustrum* and *Gyrodactylus bullatarudis* are shared amongpoeciliids. The trematode *Culuwiya cichlidorum* infected four poeciliids, plus a cichlid, while another trematode *Crassicutis cichlasomae* and the nematode *Rhabdochona kidderi* infected both species of cichlids. From an ecological perspective, all these related host species are used equally by these autogenic parasites, since variations in prevalences, mean intensities, and abundances of the above helminth species among related host species were not extensively different (Table 1). The remaining fish species, *A. mexicanus*, *R. guatemalensis*, and *S. marmoratus*, each have a particular assemblage of helminths.

The presence of specialist specific helminths in other unrelated host taxon was only occasionally recorded. Three helminth species were shared between host species of different families, *C. cichlidorum* was shared by four species of poeciliids and one of cichlid, reaching high prevalences in the poeciliids, but also attaining a rather high prevalence (26%) in the cichlid. The trematode *Genarchella isabellae* was shared by the heptapterid *R. guatemalensis* and the synbranchid *S. marmoratus*, reaching a significantly higher prevalence in the latter host species. The nematode *R. kidderi* was recorded from both cichlids but rarely from *R. guatemalensis* (Table 1).

**Figure 1.**
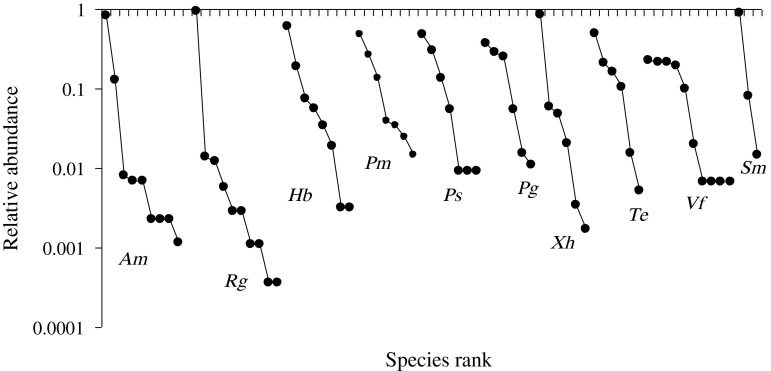
Patterns of relative abundance of 24 species of helminths in 11 component communities of freshwater fishes from Río Apazapan, Río La Antigua basin, Mexico (fish species: *Am*, *A*. *mexicanus*; *Rg*, *Rhamdia guatemalensis*; *Hb*, *Pseudoxiphophorus bimaculata*; *Pm*, *Poecilia mexicana*; *Ps*, *P*. *sphenops*; *Pg*, *Poeciliopsis gracilis*; *Xh*, *Xiphophorus helleri*; *Te*, *Thorichthys helleri*; *Vf*, *Vieja fenestrata*; *Sm*, *Sicydium gymnogaster*).

An analysis of distribution of abundances was performed including all helminth species (autogenic adults plus allogenic larvae), the numerically dominant species in *A. mexicanus*, *R. guatemalensis*, *X. helleri*, and *S. marmoratus* was the allogenic metacercariae *C. formosanus*, reaching a proportion (*P*_i_) of 0.83 to 0.95 of the total of helminth individuals recorded from these component communities. The also allogenic metacercariae *A. nana* were dominant (*P*_i_ = 0.61) in *H. bimaculata*. However, autogenic specialists were dominant in the other component communities: *C. cichlidorum* dominated in the three poeciliids, and *C. cichlasomae* was dominant in *V. fenestrata*, while *R. kidderi* dominated in *T. helleri*. To unveil the role of adult autogenic species, the analysis of distribution of abundances was performed without allogenics and other larval species. A pattern arose in which each component community was numerically dominated by a single autogenic specialist helminth (Fig. 1). The monogenean *U*. *strombicirrus* dominated the component community of *A. mexicanus*, the nematode *Cucullanus mexicanus* dominated in *R. guatemalensis*, the nematode *R. kidderi* dominated in *T. helleri*, the trematode *C. cichlasomae* dominated in *V. fenestrata*, and the trematode *G. isabellae* dominated in *S. marmoratus*. For most poeciliids, the dominant species was *C. cichlidorum*, except in *P. bimaculata* whose component community was dominated by the monogenean *U. vaginoclaustrum*.

**Figure 2.**
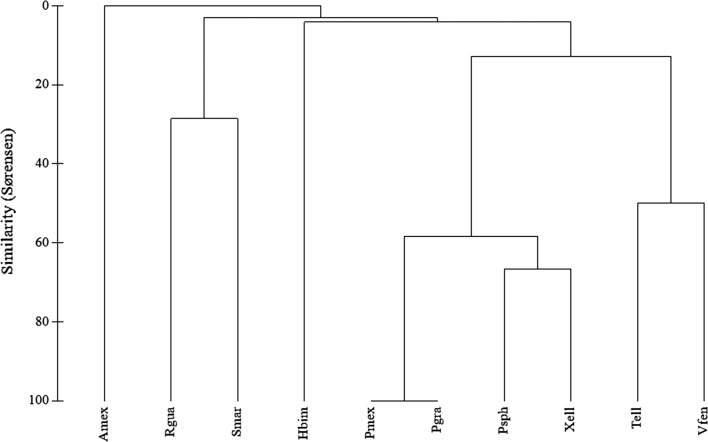
Dendrogram resulting from a similarity matrix based on the Sørensen measure for component communities of adult autogenic helminth parasites of 10 fish species from Apazapan, Río La Antigua, Veracruz, Mexico. Host species are: Amex, *A. mexicanus*; Rgua, *R. guatemalensis*; Smar, *S. marmoratus*; Hbim, *P. bimaculata*; Pmex, *Poecilia mexicana*; Pgra, *Poeciliosis gracilis*; Psph, *Poecilia sphenops*; Xell, *X. helleri*; Tell, *T. ellioti*; Vfen, *V. fenestrata*.

Analyses of similarity (Fig. 2) confirmed the sharing of species between related host taxa. Four species of poeciliids were grouped together (Sørensen’s index >60%), as well as the two cichlids that form a second group (similitude ≈50%). However, both groups, poeciliids and cichlids, displayed a comparatively low degree of similitude (<20%) between them. Minimum similarity (>>5%) or no similarity at all was recorded between all other host species, confirming each fish species hosting an almost exclusive assemblage of helminths with minimum sharing of species.

Most helminths in Apazapan fishes are host-specific since 24/36 species were recorded parasitizing a single host species. Sharing of host species was recorded in the 12 other helminth species (six allogenics and six autogenics, see Table 1). As expected, the six allogenic species behave as generalists; values of the index of specificity for these species (Table 3) point out infection of different genera of hosts in the same family (*A*. *felippei*, *P*. *minimum*, both *S*_TD_ = 1.4) or genera from different families (four allogenic species with *S*_TD_ ≥ 3.0). Four of the six autogenic adult helminth species infected two (*G. isabellae*, *C. cichlasomae*, *U. vaginoclaustrum*, *G. bullatarudis*), three (*R. kidderi*), or up to five host species (*C. cichlidorum*) in Apazapan. However, a degree of host specificity, mostly at the family level, was recorded among these species. A value of *S*_TD_ = 1 was obtained for *C. cichlasomae*, *U. vaginoclaustrum*, and *G. bullatarudis* (Table 3), showing host specificity at the host family level. An *S*_TD_ = 1.66 for *R. kidderi* was obtained because it was recorded in two host species of the same family, plus a third fish species of a different family. An equal value of *S*_TD_ = 1.66 was obtained for *C. cichlidorum* from four poeciliid and a cichlid species. A high value of the index, *S*_TD_ = 4.0, was calculated for *G. isabellae* whose two host species in Apazapan belong to fish families of different orders. The introduced metacercariae of *C*. *formosanus* also reach an *S*_TD_ = 4.0 value. The high value for the host-specificity index, reached by *G. isabellae*, *S*_TD_* = 4.0 (Table 3), reflects its asymmetrical usage of host species, its higher numbers (prevalence and abundance) in the synbranchid *S. marmoratus* as compared to its second host species, the heptapterid *R. bimaculata*. The value of *S*_TD_* was also higher in *C. cichlidorum* as compared to all other helminths, reflecting the highest prevalences of this trematode in all four poeciliids, and a lower, but also high prevalence in a cichlid *V*. *fenestrata*. The remaining four adult helminth species (Table 3) had an *S*_TD_* = 2.0 reflecting a relatively high host specificity at the host family level; where most of the recorded host species are congeners (*C*. *cichlasomae*, *U*. *vaginoclautrum*, *G*. *bullatarudis*) or hosts belong to the same family but mostly from different genera (*R*. *kidderi*). Low values of *S*_TD_* were recorded for the allogenic metacercariae *C*. *formosanus*, *P*. *minimum*, *U*. *ambloplitis*, and *Clinostomum*, thus identifying them as generalists.

**Table 3.** Values of Poulin and Mouillot’s [26, 27] indices of host specificity, *S*_TD_ and *S*_TD_* for six species of adult autogenic helminths (marked as AU) and six allogenic larval (AL) parasites of 11 freshwater fishes of Apazapan, Río La Antigua, Veracruz, Mexico (note that for all other 18 adult autogenic helminth species having only one host, *S*_TD_ = 1, and *S*_TD_* = 1).

	*S*_TD_	*S*_TD_*
*C. cichlidorum*^AU^	2.2	2.3
*G. isabellae*^AU^	4.0	4.0
*C. cichlasomae*^AU^	1.0	2.0
*Clinostomum*^AL^	2.9	1.5
*A*. *felippei*^AL^	1.4	2.3
*C*. *formosanus*^AL^	4.0	0.2
*P*. *minimum*^AL^	1.4	0.6
*U*. *ambloplitis*^AL^	3.0	1.2
*U. vaginoclaustrum*^AU^	1.0	2.0
*G. bullatarudis*^AU^	1.0	1.5
*R. kidderi*^AU^	1.7	2.0
*Contracaecum* sp.^AL^	3.0	2.6

## Discussion

Host specificity makes an important contribution to the structure of the Mexican fauna of helminth parasites of freshwater fishes. Each family of fish has their own parasites such that specialist species are comparatively more numerous than generalist species in a given locality. While most of the relatively few allogenic helminths distribute widely, infecting most sympatric fish species in a locality, each fish taxon has a particular range of autogenic specialist helminth species that are very important in structuring the local community. Patterns described by our work include high component community similarity among related host species, high prevalences and abundances of autogenic specialist species, sharing of autogenic specialist helminths largely limited to related host species, and less frequent sharing among non-related host species. We interpret this as a strategy where autogenic helminth species preferably infect a range of hosts related at the family level. This strategy enables autogenic species to find suitable hosts and to reach the population density necessary for reproduction and genetic exchange in the highly diverse tropical environments.

There is a basic community structure supported on the autogenic species, over which allogenic species are superimposed. In the context of the current investigation and our findings, we can state that all autogenics are specialist species, while allogenics behave as generalists. This statement points out an important difference when comparing helminth communities of fish from Mexican tropical latitudes versus northern temperate systems. In northern temperate systems, fish helminth communities are often dominated by acanthocephalans [17]. Acanthocephalans are autogenic species commonly found in a wide range of host taxa (i.e. they are generalists), but achieving reproductive success in a few (i.e. they behave mostly as specialists) [17]. Acanthocephalans in Mexican and Central American freshwater fishes are seldom, if ever, common [29–31], such that their absence is a crucial distinction between temperate and Neotropical fish helminth communities (Kennedy CR Pers. Com.). Interestingly and significantly there is empirical evidence that tropical fishes do have a range of autogenic specialists which explains high degrees of similarity between localities [34]. However, a metacommunity study of helminth parasites of eels in the River Exe system [16] also demonstrated the presence of a range of autogenic specialists which, in this case, did not confer similarity between localities. The difference seems to be the role of the acanthocephalans that tend to dominate freshwater fish parasite communities at northern temperate latitudes [16, 17] and that are very uncommon and generally occur at low prevalence and abundance levels, and certainly do not dominate tropical assemblages [34 and present work].

Our results shed light on the formation and development of the tropical communities of helminth parasites of freshwater fishes, and give additional support to the idea that the distribution of the species of helminths follows that of their host families [29–31]. When a colonizing fish arrives in a new habitat, it brings its parasites. Host-sharing will most often be successful only if a related host species is found previously established in this new body of water. Otherwise, parasites will continue to parasitize only the host they arrived with [25]. In the same way, the new arriving host can receive additional parasites only from related hosts previously inhabiting the locality. Host specificity simultaneously limits the ability of both host and parasite to form new systems [25].

The present results show that helminths in Apazapan fishes achieve high abundances in closely related host species. These high parasite densities seem to be the rule in Neotropical regions and these results are supported by those of [32, 33], who found high densities in fishes of other Mexican basins. Availability and effective use of resources seem to promote high densities of parasites, improving reproductive efficiency. This in turn can promote a high production of parasite propagules favoring transmission, host colonization, and further dispersion. These results suggest that the more host species are related, the greater the probability that the parasite population will persist and spread [23, 25].

We interpret that host specificity to the host family level leads to an efficient approach given the wide resource availability for freshwater fish parasites in the tropics. Specialist parasites must have acquired a number of adaptations favoring encounters and invasion possibilities with its definitive fish hosts [11, 15]. This would counterbalance the dilution effect related to high ichthyological diversity in tropical environments. A generalist approach of helminths in tropical bodies of water would lead to a dispersion of parasite individuals among the many unrelated available hosts, thus effectively diluting the possibilities of reproduction and of genetic interchange. Placing fewer parasite individuals in many hosts could involve a disadvantage when trying to attain an optimal concentration of individuals for parasite reproduction [11]. Similarly, very narrow host specificity, to the species level perhaps, could lead to parasites facing a shortage of resources, because fish in the tropics often live immersed in a matrix of many sympatric fish species, some of them related, but many more not at all. Infecting related hosts in tropical environments can guarantee good opportunities for host colonization, and might guarantee enough resources for parasites and good chances for attaining optimal population densities for parasite reproduction. Greater specialization on related host species is an advantage that links the fates of parasites to that of their hosts, and provides highly host-specific parasites a good opportunity to disperse.

It is worth noting that some fish populations, such as the poeciliid *Pseudoxiphophorus bimaculata* in the upper Río La Antigua basin, have developed a range of specialist helminth species only found with *P*. *bimaculata* in that geographical area. Helminth parasites of *P*. *bimaculata* have regularly been reported from several populations of this host species in Mexican basins [29 and references herein]. Considering this, we can be relatively confident that helminth species that parasitize this host species in Apazapan are specialist helminths and indeed, endemic to this basin. The absence of exchange of parasites between *P*. *bimaculata* and the other four sympatric poeciliids in Apazapan is striking. Most poeciliids have similar morphologies, feeding habits, and similar modes of life, i.e. the four species of poeciliids inhabiting Arroyo Apazapan have similar ecological characteristics [19–21]. The different content of parasites recorded for sympatric poeciliid fishes in Apazapan suggests that the narrow specificity observed for helminths of *P*. *bimaculata* could have a phylogenetic basis, and could reflect the phylogeny of both the host and the parasites. Testing such a hypothesis requires further work.

A discussion of local, general, or regional characterization of helminth host specificity is required. Taking into account the entire known host record of occurrence of each of the helminth species recorded from Apazapan fishes, we can confirm the identity of their preferred hosts, as well as carry out an assessment of the degree of specific or generalistic behavior exhibited by the particular species in Apazapan. Acknowledging that the use of larval forms could add noise to host-specificity analyses because in this context the term host should refer only to those species in which a parasite can successfully survive and grow [23–27], all helminth larval forms were excluded from this comparison. Remarkably, the autogenic adult nematode *Procamallanus neocaballeroi*, a specialist in Apazapan *S*_TD_ = 1, could be considered generalist, *S*_TD_ = 2.83, when the total record of two fish families, three genera, and three species [see 4] is taken into consideration. The trematode *Magnivitellinum simplex* was also qualified in Apazapan as specialist (*S*_TD_ = 1), but is considered generalist (*S*_TD_ = 3.3) when its total host record (three orders, four families, four genera, six species; [29]) is considered. As a consequence, a particular qualification of a species as generalist/specialist based on local data does not necessarily hold true when accounting for the entire host record for the species, and vice versa. This is very important because it is widely accepted that the specialist/generalist character is a property of species of parasites, meaning that the species is either a specialist or a generalist and cannot be one at a local scale and another at a regional scale. Our results show that even generalist species (such as *M*. *simplex* or *P*. *neocaballeroi*) cannot invade all fish, and cannot be exchanged evenly among sympatric host species. Thereby, our results suggest caution when evaluating specificity using bibliographical records.

While the patterns described in this paper seem to be consistent among freshwater fish helminth communities in Neotropical Mexico, more comparable studies are needed to evaluate the generality of these results.
